# Supplementary figures and images for: Quantitative imaging of loop extruders rebuilding interphase genome architecture after mitosis
Source: J Cell Biol. 2025 Jan 9;224(3):e202405169. doi: 10.1083/jcb.202405169 (PMC11716112; doi:10.1083/jcb.202405169)

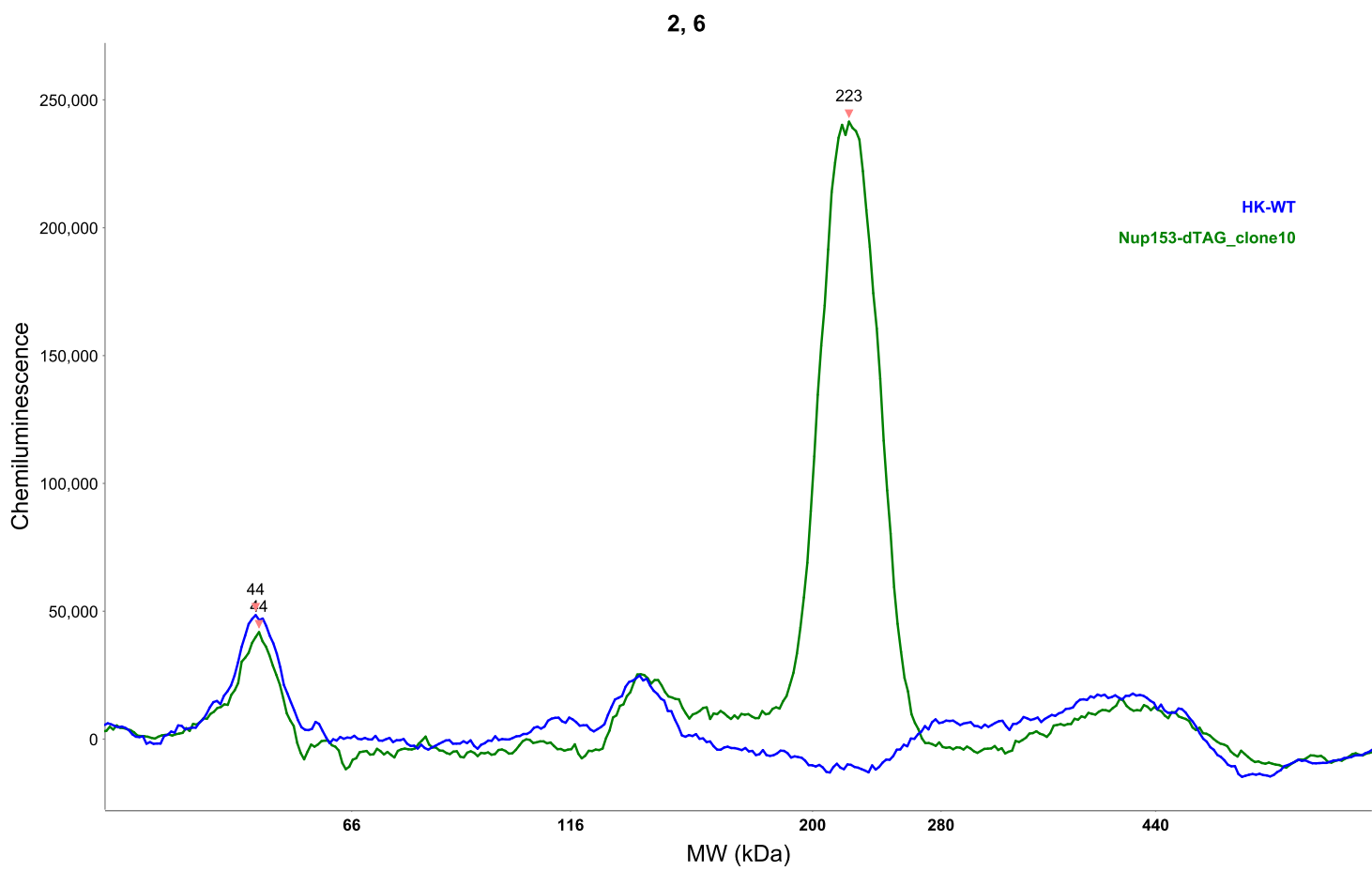

10, 11

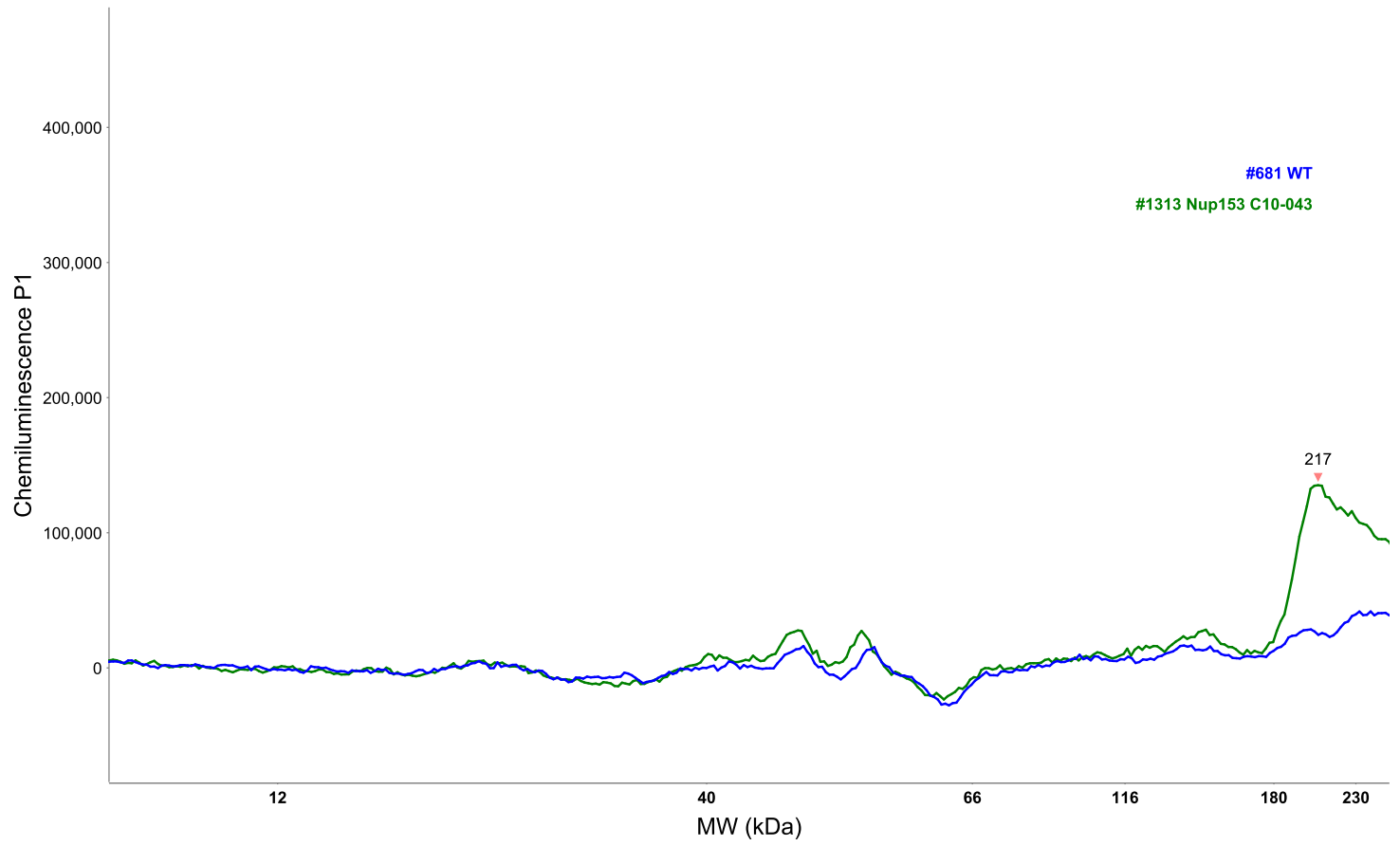

14, 18

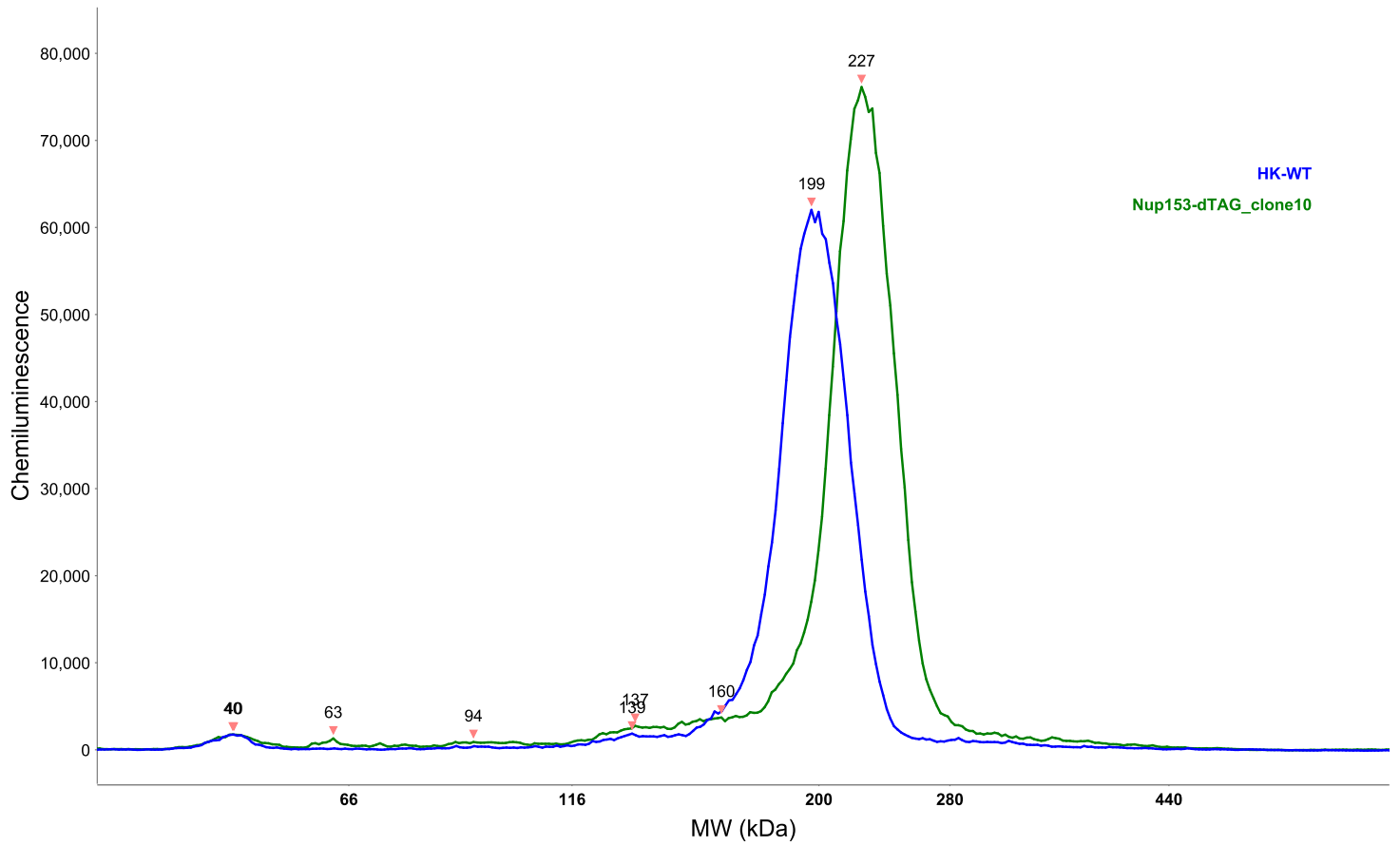

Supplement: SourceData F2 — is the source file for Fig. 2. [file jcb_202405169_sourcedataf2.pdf]

# anti-CTCF

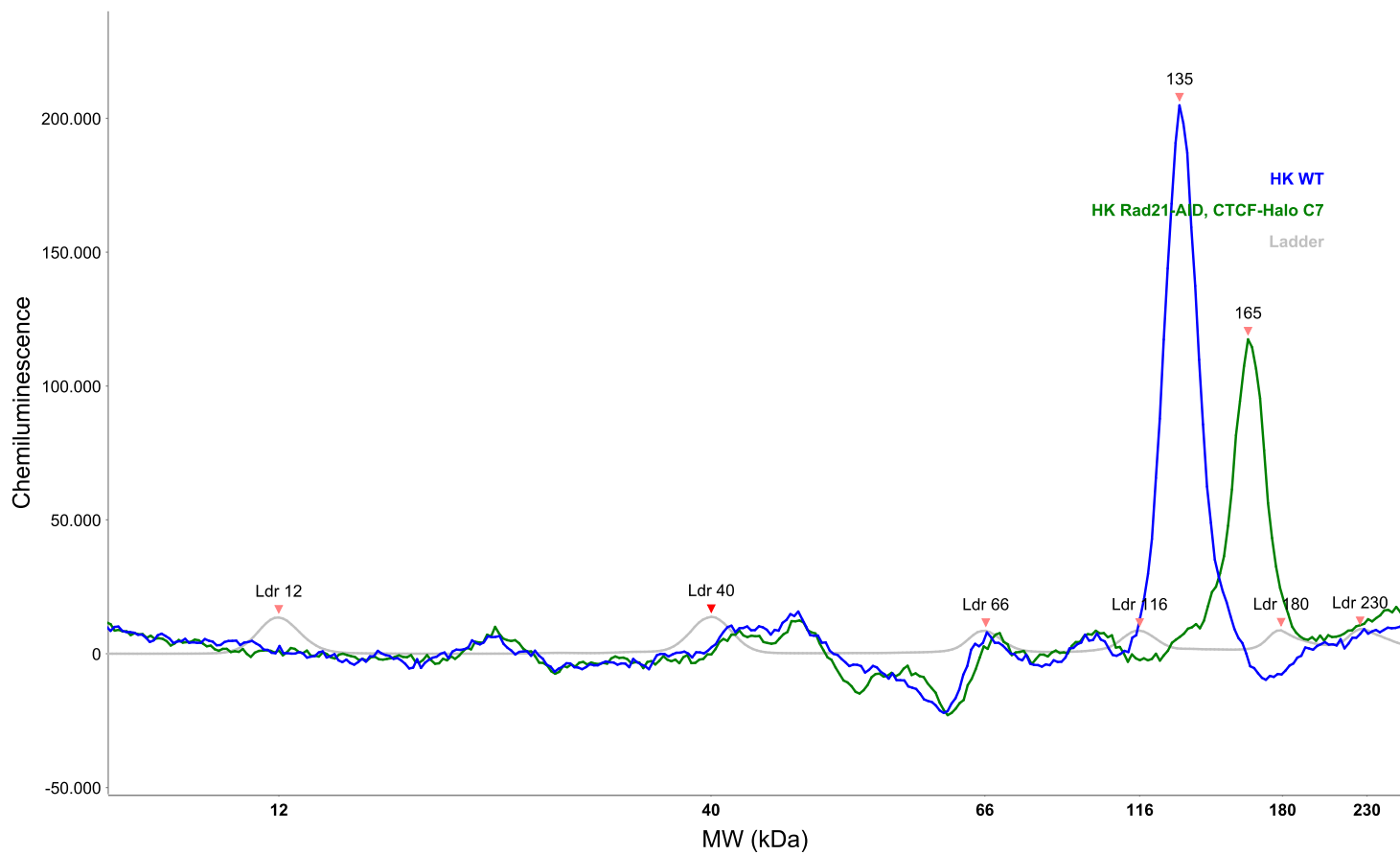

anti-Halo

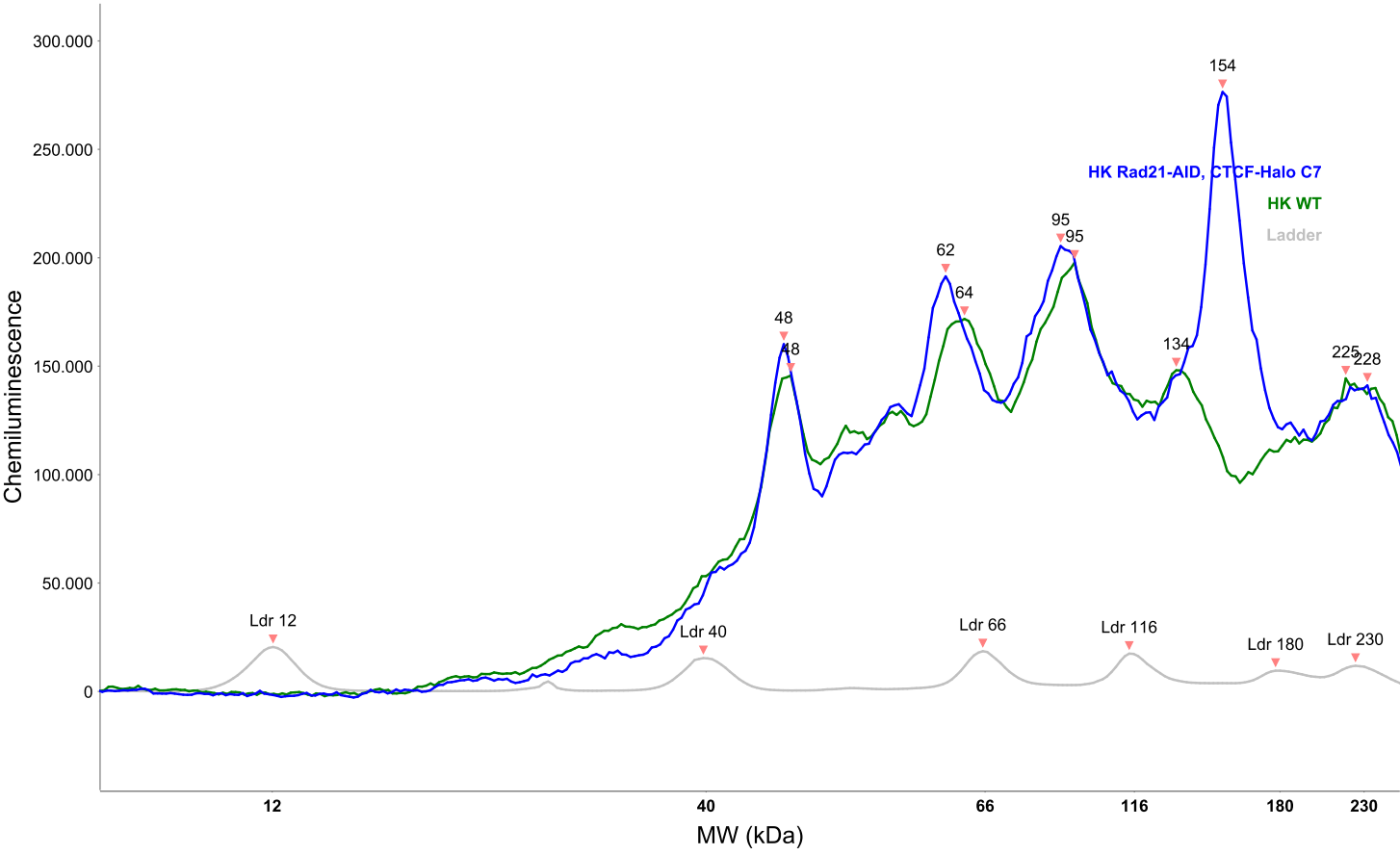

anti-Rad21

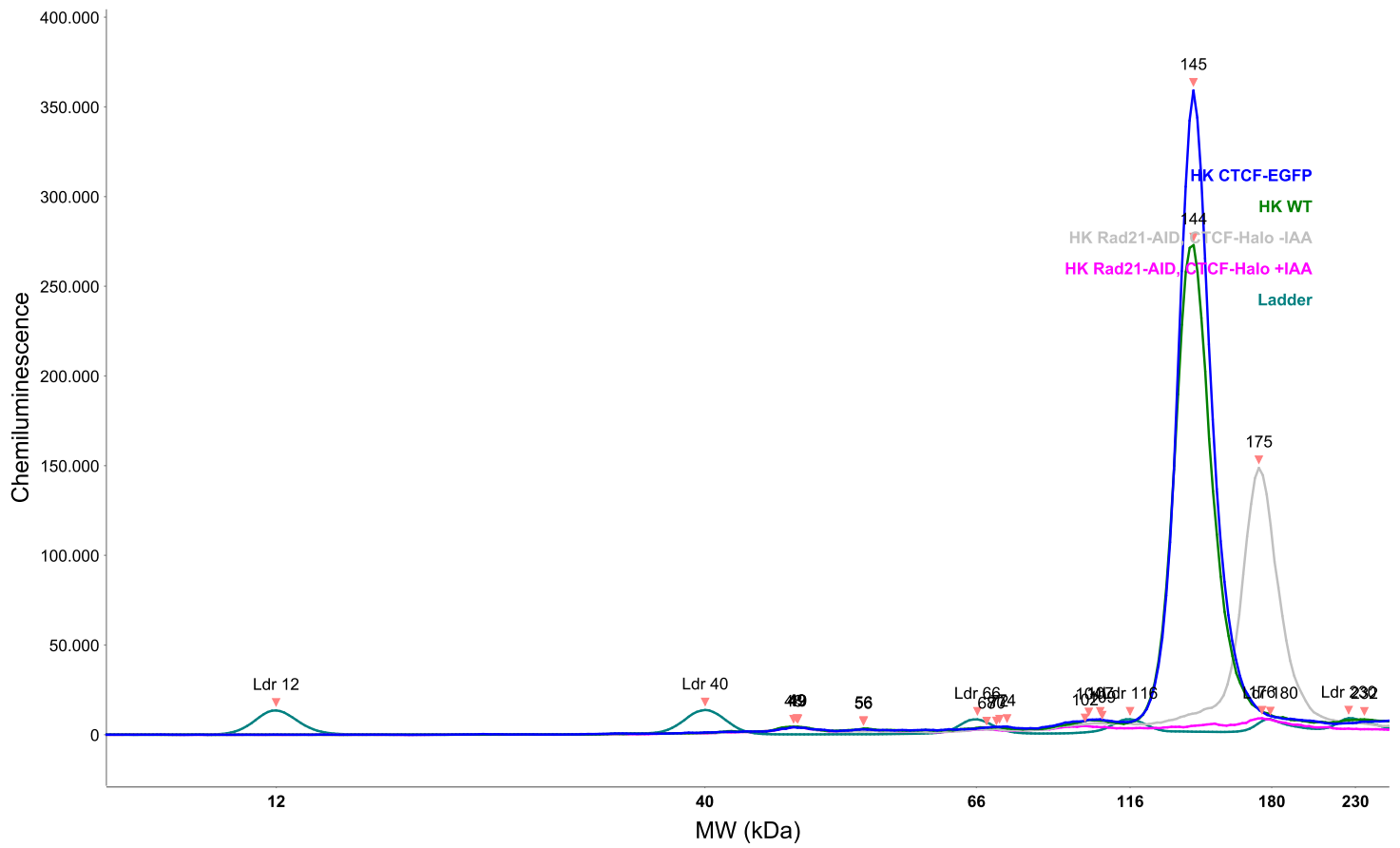

Supplement: SourceData FS4 — is the source file for Fig. S4. [file jcb_202405169_sourcedatafs4.pdf]
